# Long-term analysis of chronic pain associated with lower extremity injuries

**DOI:** 10.1007/s00402-022-04717-6

**Published:** 2022-12-01

**Authors:** Thomas Rauer, Eva Friedl, Jamison G. Gamble, Boris A. Zelle, Hans-Christoph Pape, Roman Pfeifer

**Affiliations:** 1 Department of Trauma Surgery, University Hospital Zurich, 8091 Zurich, Switzerland; 2 St. George’s University School of Medicine, St. George, Grenada; 3 Department of Orthopedics, University of Texas Health Science Center at San Antonio, San Antonio, TX USA

**Keywords:** Long-term outcome, Lower limb, Multiple injuries, Acetabulum, Chronic pain, Polytrauma

## Abstract

**Introduction:**

The main objective of this study is to examine chronic pain and limping in relation to lower extremity and pelvic fracture location in addition to fracture combinations if multiple fractures are present on the same leg that have not been previously reported. We hypothesize that fracture pattern and location of lower extremity and pelvis fractures of multiple injured patients influence their long-term pain outcome.

**Materials and methods:**

Retrospective cohort study. Patients with treated multiple lower limb and pelvic fractures at a level 1 trauma center and followed up for at least 10 years postinjury were assessed. Lower leg pain subdivided into persistent, load-dependent and intermittent pain, as well as limping were recorded by using self-administered patient questionnaires and standardized physical examinations performed by a trauma surgeon. Descriptive statistics were used to present comparative measurements between groups.

**Results:**

Fifty-seven percent of patients (*n* = 301) showed chronic lower limb pain 10 years postinjury. Ten percent of all patients with chronic pain displayed persistent pain, and here the most common fracture combination was tibial shaft fractures in combination with femoral shaft or proximal tibial fractures (13%). One hundred fifty-one patients reported load-dependent pain, with the most common fracture combinations being fractures of the foot in combination with femoral shaft fractures or distal tibial fractures (11%). One hundred twenty patients reported intermittent pain, with the most common fracture combinations involving the shaft of the tibia with either the femoral shaft or distal tibia (9%). Two hundred fifteen patients showed a persistent limp, and here the most common fractures were fractures of the femoral shaft (19%), tibial shaft (17%), and pelvis (15%).

**Conclusions:**

In multiple injured patients with lower extremity injuries, the combination of fractures and their location are critical factors in long-term outcome. Patients with chronic persistent or load-dependent pain often had underlying femoral shaft fractures in combination with joint fractures.

## Introduction

Due to advances in prehospital care, modern intensive care concepts, formation of specialized trauma centers, and improved surgical management, the survival rate of severely injured patients has increased from 60 to 85–88% in recent decades [1, 2]. With increased survival rates, the long-term functional outcomes of polytrauma patients have gained importance [3]. Specifically, lower extremity injuries have been associated with significant functional impairments and low satisfaction scores in polytrauma patients [4–7]. A detailed investigation of long-term outcome and their influencing factors after lower extremity injury was performed within the Lower Extremity Assessment Project (LEAP) [8–11].

The impact of lower extremity injuries on outcome has also already been described by our group [12]. It has been reported that fractures below the knee are frequently associated with persistent functional deficits in trauma patients [13]. It was also suggested that not only the individual fracture but more importantly fracture patterns influence long-term outcome [13]. Furthermore, it was emphasized that delayed treatment, a thin soft tissue envelope below the knee, high energy trauma, unfavorable blood supply and complex fracture patterns may contribute to unfavorable outcomes [13]. However, studies providing detailed information on the impact of the injury pattern of lower extremity and pelvic fractures on the functional outcomes in polytrauma patients remain limited. Therefore, the goal of this study is to examine the relationship between the injury pattern of lower extremity fractures and the functional long-term outcomes in polytrauma patients. The main objective of this study is to identify chronic pain associated with lower extremity injury patterns that have not been previously reported. We hypothesize that fracture pattern and location of lower extremity and pelvis fractures of multiple injured patients influence their long-term pain outcome.

## Materials and methods

We performed a retrospective cohort study of prospective acquired data involving patients with multiple fractures of the lower limb and pelvis that were initially treated in a Level 1 Trauma center and followed up for a minimum of 10 years postinjury. The standardized follow-up evaluation was performed by a trauma surgeon. All Patients provided written consent and the study protocol was approved by the local Institutional Review Board (No. 2326-2000/03/22).

Patients treated between 01/01/1973 and 12/31/1990 were retrospectively reviewed. If inclusion and exclusion criteria were met, patients were invited for physical examinations. Further details of patient recruitment and measures against biases were previously published [14, 15]. Out of the initial cohort (*n* = 637) 525 patients met the inclusion criteria: age 3–58 years at the time of injury, multiple injured patients and associated fractures to the lower limb and/or pelvis.

Influencing variables were obtained using the patient charts including: Fracture location, patients age at the time of injury, gender, cause of injury and injury severity measured by Abbreviated Injury Score (AIS) [16]. Target variables were obtained using self-administered patient questionnaires as well as standardized physical examinations of all injured locations as performed by a trauma surgeon. The following functional variables were recorded: lower leg pain subdivided into persistent pain, load-dependent pain and intermittent pain, as well as limping.

Accordingly, the patients were divided into subgroups.

Within these subgroups, the most common fracture combinations were obtained. The rest were categorized in a separate cohort defined as “other.”

### Definitions

- Fracture localization was classified according to the OTA/AO classification system [17].
- Injury severity was classified in accordance with the Abbreviated Injury Scale (AIS) [18], ranging from 0 (no injury) to 6 (unsurvivable). We used the maximum AIS score (MAIS) [19] for lower extremity injuries as a measure of injury severity.
- Intraarticular knee fractures were divided in the subcategories: fracture of the proximal tibia or fracture or the distal femur.

### Outcome parameters

- Persistent pain was defined as self-reported daily pain [12].
- Load-dependent pain was documented if patients experienced pain subsequent to physical activities (e.g., pain with walking or activities of daily living).
- Intermittent pain was distinguished from the previous two groups.
- Limping was evaluated in gait analysis and physical examination. Gait abnormalities were assessed by the evaluating trauma surgeon and were recorded as limp versus no limp.

### Statistical analysis

The primary analysis of this paper is based on descriptive statistics to present comparative measurements between groups. Continuous variables are presented as mean with standard deviation (SD), categorical variables as numbers and percentages. Statistical analysis was performed using SPSS for Windows (SPSS Inc., Chicago, IL). Results were considered to be significant when *p* value was < 0.05.

## Results

A total of 637 multiple injured patients were examined 10 years after injury. A total of 525 had lower limb and/or pelvis injuries and therefore were included in this analysis. The average age at the time of injury was 26 years (SD 11.7 years) with a range from 3 to 58 years. The male-to-female ratio in total was approximately 3 to 1 (male *n* = 395). The average total number of injuries was 4.5 (SD 1.9). The average MAIS for lower extremity injuries was 2.8 (SD 0.5) with a range from 1 to 4.

More than half of the study cohort still demonstrated lower limb pain 10 years postinjury (*n* = 301; 57.3%). This most commonly manifested as load-dependent pain (*n* = 151; 50.2%), followed closely by intermittent pain (*n* = 120; 39.9%). Persistent lower limb pain was seen in 9.9% (*n* = 30).

**Fig. 1:**
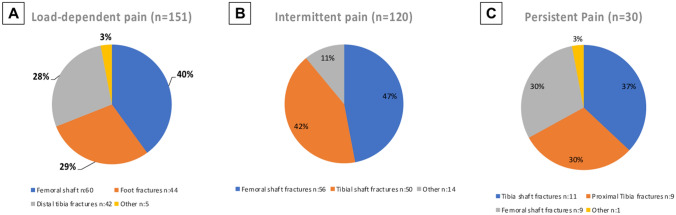
Most common fractures of all patients with long-term pain in multiple fractures of the lower limbs presenting as **A** load-depending pain, **B** intermittent pain, **C** persistent pain

**Fig. 2:**
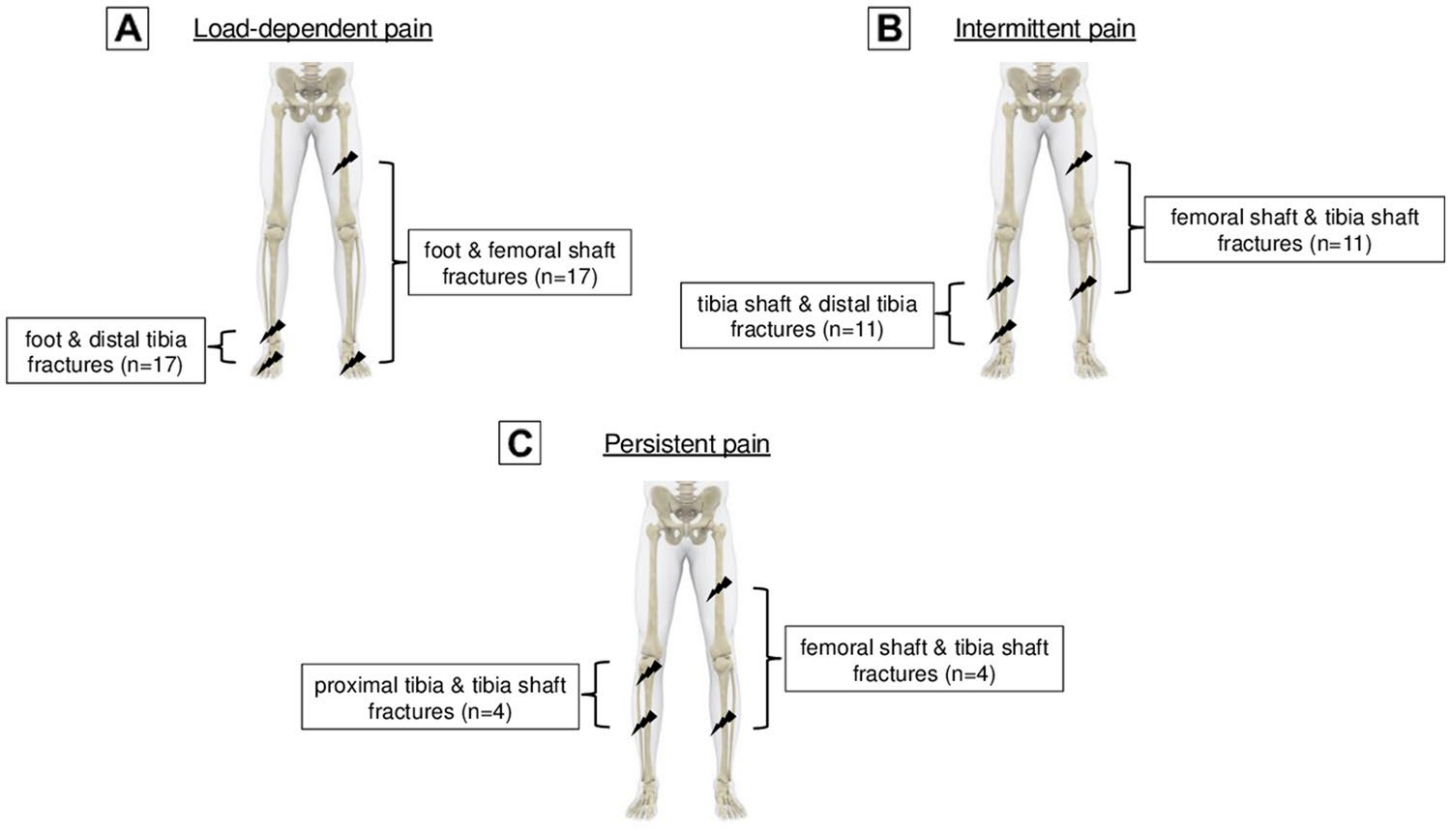
Most common fracture combination of all patients with long-term pain in multiple fractures of the lower limbs presenting as **A** load-depending pain, **B** intermittent pain, **C** persistent pain

Load-dependent pain was most prevalent at 50% and was most common in patients with femoral shaft fractures (40%; *n* = 60), foot fractures (29%; *n* = 44) and distal tibia fractures (28%; *n* = 42) (Fig. 1A). The most common fracture combinations were fractures of the foot combined with femoral shaft fractures or distal tibia fractures. Each making up 11% (*n* = 17) of the group cohort (Fig. 2A).

Intermittent pain was present in 40% of all patients with pain with multiple lower limb fractures. Out of these, 47% (*n* = 56) presented with a femoral shaft fracture and 42% (*n* = 50) a tibia shaft fracture (Fig. 1B). Fracture combinations involving the shaft of the tibia with either the femoral shaft or distal tibia occurred most frequent with at 9% each, respectively (*n* = 11) (Fig. 2B).

Persistent pain was still present in 10% of all patients with multiple injuries of the lower extremity. Tibia shaft fractures (37%; *n* = 11), proximal tibia fractures (30%; *n* = 9) and femoral shaft fractures (30%; *n* = 9) predominated this group (Fig. 1C). The combination of a fracture of the tibial shaft with either the femoral shaft or proximal tibia was most common at 13% (*n* = 4) (Fig. 2C).

Forty-one percent of patients still showed a persistent limp 10 years postinjury (*n* = 215). Of those demonstrating a persistent limp, a one-sided limp was seen in 65% of cases (*n* = 140), while a bilateral limp was seen in 35% of cases (*n* = 75). The most common fractures resulting in a persistent limp involved fractures of the femoral shaft (*n* = 41; 19%), fractures of the tibia shaft (*n* = 37; 17%) and fractures of the pelvis (*n* = 32; 15%).

## Discussion

Identifying and analyzing risk factors influencing the long-term outcomes can produce important information for establishing new treatment strategies and guidelines improving long-term functional results.

Our study revealed the following results:

1. The combination of fractures and their location are determining factors for the long-term outcome in polytrauma patients with lower extremity injuries
2. Chronic persistent or load-dependent pain often had underlying femoral shaft fractures in combination with joint fractures.

The degree of soft tissue injury is certainly a crucial contributory factor affecting long-term outcome in patients with lower extremity injuries. Thus, the studies of the Lower Extremity Assessment Project (LEAP) described factors that influence the long-term outcome of limb-threatening lower extremity injuries and showed that patients with amputations had comparable functional outcomes to patients with reconstructions [8–11]. However, information about the long-term pain outcome of patients with multiple fractures of the lower limb and pelvis is sparse.

Continuous pain is very frequently reported following multiple trauma of the lower extremity. This is in accordance with recent studies showing a significant impact on functional recovery of polytrauma patients with fractures below the knee [13]. Persistent hip pain is known in patients with a history of femoral shaft fractures [20]. Possible attributions to cause continuous pain is osteoporosis due to immobility [21] and the development of arthritis [22–24].

Chronic persistent and load-dependent pain were both frequently associated with femoral shaft fracture in combination with articular fractures. Studies differentiating between stress induced and continuous pain after femoral shaft fracture are scarce. Heel pain after weight bearing can be caused by tarsal tunnel, nerve entrapment or Achilles tendinopathy as a result of soft tissue damage [25]. Foot fractures can be more easily overlooked in the initial diagnostics of a polytrauma patient. Delayed treatment can cause chronic pain.

Studies suggest the presence of intra-articular hip pathology in patients with chronic pain following femoral shaft fractures, which may provide a possible explanation as to why this fracture type is the most common fracture overall in our study involving patients exhibiting any chronic or stress induced pain [20]. All fractures healed while nonunion was not a relevant factor. Heterotopic ossification may result in limited mobility, thereby causing weight bearing chronic pain. Several studies show a higher rate of heterotopic joint ossification in ventilated patients or patients with a head injury, which is a common occurrence in patients of polytrauma [26–28]. Furthermore, muscle weakness due to immobility can be attributed as a cause of stress induced pain [29, 30].

A remarkable 41% of patients still exhibited a persistent limp 10 years post trauma. This demonstrates the value in the examination of gait abnormalities, especially when considering that one of the primary factors influencing patient satisfaction is extremity function [31]. Long bone shaft fractures were shown to be the most common cause of an abnormal gait in patients.

Pelvic ring instability is a known cause of gait abnormality [32]. Research on long-term gait abnormality for long bone fractures is scarce. It is suggested that gait abnormality after femoral shaft fractures may also be due to intraarticular hip pathologies such as labral tears or osseous bumps of the femoral neck [20]. A difference in length between both legs resulting from long bone shaft fractures could possibly also explain the cause of limping [32].

Abnormal gait can be caused by immobility of the joints following muscle contracture or pain disorder [33]. Heterotopic ossification also generates restriction in mobility [26]. Additionally, head injuries can also lead to heterotopic ossification [27, 28] which are common in polytrauma patients. High rates are also noted in ventilated patients [26].

### Limitations and strengths

The retrospective evaluation to find eligible patients is a weakness in our otherwise prospective acquired clinical study. This study is based on the retrospective analysis of data prospectively collected and documented during routine clinical practice and is therefore subject to all limitations of retrospective data analyses. Data generation is directly dependent on the accuracy of documentation, which is why data quality may be reduced for parameters that were recorded according to exclusion criteria. This may affect the generalizability of the results. As the extent of the soft tissue injury was not documented during database generation, we cannot make any statements about the impact of the soft tissue damage on the long-term pain outcome. Strengths of our study include a large cohort and long-term follow-up of at least 10 years, exclusive examination and consultation of patients by a trauma surgeon.

## Conclusion

In multiple injured patients with lower extremity injuries, the combination of fractures and their location are critical factors in long-term outcome. Patients with chronic persistent or load-dependent pain often had underlying femoral shaft fractures in combination with joint fractures.

Patients with the types of injury patterns described here may benefit from structured and targeted long-term physiotherapy to prevent the development of chronic pain.

## Data Availability

The datasets are accessible on reasonable request.
